# Development of a Prediction Model for Carbapenem-Resistant Enterobacterales Acquisition in Liver Transplant Recipients

**DOI:** 10.1017/ash.2025.354

**Published:** 2025-09-24

**Authors:** Mijung Kim, JaHyun Kang

**Affiliations:** 1 Seoul National University, College of Nursing/ Asan Medical Center; 2 Seoul National University College of Nursing

## Abstract

**Background:** Carbapenem-resistant Enterobacterales (CRE) are significant healthcare-associated pathogens. Liver transplant (LT) recipients are particularly vulnerable to CRE acquisition due to frequent hospitalizations, extensive antibiotic exposure, and prolonged stays in intensive care units. This study aimed to develop and evaluate prediction models for CRE acquisition in LT recipients at a hospital where more than 500 LT surgeries are performed annually. **Method:** This case-control study retrospectively analyzed the electronic medical records of 1,250 adult LT recipients (250 CRE-positive and 1,000 CRE-negative cases) at a 2,768-bed tertiary hospital in Seoul, Korea, from February 2020 to February 2024. Data imbalance was addressed using the synthetic minority over-sampling technique, and missing values were handled through median imputation and k-nearest neighbor imputation methods. Prediction models were developed using logistic regression, random forest, and extreme gradient boosting (XGBoost) algorithms, with optimal models selected through 5-fold cross-validation and recursive feature elimination. Model interpretability was enhanced using Shapley additive explanations and partial dependence plot analyses. **Result:** Of the CRE isolates, 94% were carbapenemase-producing Enterobacterales, with Klebsiella pneumoniae comprising 55.7% of all CRE isolates. Univariate analysis revealed significant differences between groups in LT month (June-September, p<.001), mechanical ventilation over 72 hours (p=.002), and model for end-stage liver disease (MELD) score (p=.041). The XGBoost model, selected as the final model, demonstrated strong specificity (0.848) and a high negative predictive value (NPV 0.830) for identifying non-carriers, although its overall predictive power was limited. Features used in the XGBoost model included LT month, third-generation cephalosporins, and the presence of hepatocellular carcinoma, all of which showed a positive correlation with CRE acquisition. In contrast, mechanical ventilation over 72 hours and living donor LT exhibited negative correlations. Viral hepatitis and body mass index were included in the model, but their impact on CRE acquisition risk remained unclear. Notably, the negative association of mechanical ventilation contrasts with findings from previous studies, highlighting the need for further investigation. **Conclusion:** This study demonstrates the clinical relevance of machine learning models in predicting CRE acquisition among LT recipients. The XGBoost model showed high specificity and NPV, indicating its potential to effectively identify low-risk patients. Future studies could benefit from adopting prospective, multicenter designs to clarify causal relationships and improve model performance.

